# Construction and validation of a musculoskeletal disease risk prediction model for underground coal miners

**DOI:** 10.3389/fpubh.2023.1099175

**Published:** 2023-07-11

**Authors:** Haili Zhao, Hong Dou, Xianting Yong, Wei Liu, Saiyidan Yalimaimaiti, Ying Yang, Xiaoqiao Liang, Lili Sun, Jiwen Liu, Li Ning

**Affiliations:** ^1^College of Public Health, Xinjiang Medical University, Urumqi, China; ^2^Xinjiang Uygur Autonomous Region Third People’s Hospital, Urumqi, China; ^3^The Fifth Affiliated Hospital, Xinjiang Medical University, Urumqi, China

**Keywords:** underground coal miners, occupational stress, occupational job burnout, depression, musculoskeletal disorders, nomogram

## Abstract

**Objective:**

To understand the prevalence among underground coal miners of musculoskeletal disorders (MSDs), analyze the risk factors affecting MSDs, and develop and validate a risk prediction model for the development of MSDs.

**Materials and methods:**

MSD questionnaires were used to investigate the prevalence of work-related musculoskeletal disorders among 860 underground coal miners in Xinjiang. The Chinese versions of the Effort-Reward Imbalance Questionnaire (ERI), the Burnout Scale (MBI), and the Self-Rating Depression Inventory (SDS) were used to investigate the occupational mental health status of underground coal miners. The R4.1.3 software cart installation package was applied to randomly divide the study subjects into a 1:1 training set and validation set, screen independent predictors using single- and multi-factor regression analysis, and draw personalized nomogram graph prediction models based on regression coefficients. Subject work characteristic (ROC) curves, calibration (Calibrate) curves, and decision curves (DCA) were used to analyze the predictive value of each variable on MSDs and the net benefit.

**Results:**

(1) The prevalence of MSDs was 55.3%, 51.2%, and 41.9% since joining the workforce, in the past year, and in the past week, respectively; the highest prevalence was in the lower back (45.8% vs. 38.8% vs. 33.7%) and the lowest prevalence was in the hips and buttocks (13.3% vs. 11.4% vs. 9.1%) under different periods. (2) Underground coal miners: the mean total scores of occupational stress, burnout, and depression were 1.55 ± 0.64, 51.52 ± 11.53, and 13.83 ± 14.27, respectively. (3) Univariate regression revealed a higher prevalence of MSDs in those older than 45 years (49.5%), length of service > 15 years (56.4%), annual income <$60,000 (79.1%), and moderate burnout (43.2%). (4) Binary logistic regression showed that the prevalence of MSDs was higher for those with 5–20 years of service (OR = 0.295, 95% CI: 0.169–0.513), >20 years of service (OR = 0.845, 95% CI: 0.529–1.350), annual income ≥$60,000 (OR = 1.742, 95% CI: 1.100–2.759), and severe burnout (OR = 0.284, 95% CI: 0.109–0.739), and that these were independent predictors of the occurrence of MSDs among workers in underground coal mine operations (*p* <  0.05). (5) The areas under the ROC curve for the training and validation sets were 0.665 (95% CI: 0.615–0.716) and 0.630 (95% CI: 0.578–0.682), respectively, indicating that the model has good predictive ability; the calibration plots showed good agreement between the predicted and actual prevalence of the model; and the DCA curves suggested that the predictive value of this nomogram model for MSDs was good.

**Conclusion:**

The prevalence of MSDs among workers working underground in coal mines was high, and the constructed nomogram showed good discriminatory ability and optimal accuracy.

## 1. Introduction

The U.S. Department of Labor defines work-related musculoskeletal disorders (MSDs) as injuries or diseases of muscles, nerves, tendons, joints, cartilage, and intervertebral discs associated with exposure to risk factors in the workplace (1). These diseases not only affect the quality of life of workers but also impose a significant economic burden on society (2). Musculoskeletal disorders affect millions of European workers and are the leading health problem at work in the European Union. China is one of the richest countries in the world in terms of coal resources, with diverse geological formations, complex mining conditions, and a wide albeit uneven distribution of resources, with more coal in the north and west and less in the south and east of the country (3). In the coal mining industry, MSDs have become one of the main problems affecting the health of coal miners due to the complex mining conditions, harsh working environment, poor working conditions, and high physical load of workers (4). It is known that patients with MSDs in occupational groups often have psychological problems (5). But few scholars have focused on the effects of occupational stress, occupational job burnout, and depression on MSDs in coal miners, and to our knowledge, there are no prediction models on the risk of MSDs in underground coal miners (6–8). A comprehensive understanding of MSDs could provide insights into the factors of MSDs for early identification of high-risk groups. This could be used to effectively predict the occurrence of MSDs and provide early intervention, thereby effectively reducing the prevalence and mortality of MSDs in coal miners. Therefore, this study aimed to understand the prevalence and influencing factors of MSDs in underground coal miners in Xinjiang, China, and to establish a personalized nomogram to provide a theoretical basis for the targeted health promotion of underground coal miners.

## 2. Materials and methods

### 2.1. Object of the study

In this study, 950 underground coal miners working at two coal mining companies in Urumqi and Kashgar (two cities in Xinjiang, China) were randomly selected from August to October 2021 using the whole group sampling method. (1) Inclusion criteria: those who had worked for 1 year or more, and those who had not taken antidepressants and other drugs within the last week. (2) Exclusion criteria: those with serious organic diseases, and those with psychiatric or genetic histories. In total, 950 questionnaires were distributed, and 860 valid questionnaires were returned, for an effective rate of 90.53%.

### 2.2. Research methodology

#### 2.2.1. Socio-demographic characteristics

General demographic characteristics of the workers were collected, such as age, length of service, education, job type, shift status, annual income, smoking, and drinking.

#### 2.2.2. Occupational tension questionnaire

The Chinese version of the Effort-Reward Imbalance Questionnaire (ERI) was used to evaluate the current situation of occupational tension among coal miners. ERI > 1 means high pay, low reward; ERI = 1 refers to a balanced pay–reward state; ERI < 1 means low pay, high reward; and the larger the ratio of ERI, the higher the level of occupational tension. The total score of work engagement is such that the higher the score of the total entry, the heavier the workload.

#### 2.2.3. Burnout situation questionnaire

The Maslach Burnout Inventory (MBI), revised by Yongxin Li, was used to evaluate the current situation of burnout among coal miners. The critical values of the three latitudes of the scale were 25, 11, and 16, and burnout was divided into four levels based on these critical values: zero burnout (the scores of all three latitudes were less than the critical values), mild burnout (the scores of any one latitude were greater than or equal to the critical values), moderate burnout (the scores of any two latitudes were greater than or equal to the critical values), and high burnout (the scores of all three latitudes were greater than the critical values).

#### 2.2.4. Depression status questionnaire

The Self-Rating Depression Scale (SDS) was adopted from the Center for Epidemiologic Studies Depression Scale developed by Sirodff in 1977. Generally, a score of 16 is used as the threshold value. A total score ≥ 16 indicates depressive symptoms, which are divided into four degrees according to the score: no depressive symptoms (<16), mild depression (16–19), moderate depression (20–23), and major depression (>23).

#### 2.2.5. Occupational skeletal disorders scale

The Dutch Musculoskeletal Disorders Questionnaire (MSDs) was used. MSDs were considered to have developed when the disease lasted for more than 24 consecutive hours and MSD symptoms were present in one of the nine sites.

#### 2.2.6. Quality control

After the completion of the questionnaire and a unified review by the investigators, invalid data such as missing answers, wrong answers, etc. were eliminated and the data were carefully checked for double entries in the database.

### 2.3. Analysis methods

The data were entered into the Epidata 3.0 database, and SPSS 25.0 software was used to organize and analyze the data. The measurement data were statistically described by X ± S when conforming to the normality test. A *t*-test was used for comparison of means between two groups. One-way ANOVA was used for comparison of means between multiple groups, and the LSD method was used for a two-way comparison of means between groups. The measurement data were described by [*n* (%)], and the Chi-squared test was used for the comparison of rates. Factors with *p* < 0.05 in the one-way factor analysis of variance were included in the binary logistic regression equation. Factors with *p* < 0.05 in the binary logistic regression analysis were used for forest plotting using Graphpad prism 9.0, and for the construction and evaluation of a nomogram using R studio 4.1.3. The test level α = 0.05.

## 3. Results

### 3.1. Study participants

The study population comprised 860 underground coal mine workers, including 430 in the training set and 430 in the validation set Table 1.

**Table 1:** General demographic characteristics among underground coal miners [*n* (%)].

Factors	Total (*n* =860)	Train (*n* =430)	Validation (*n* =430)	*χ^2^*	*P* value
**Age(years)**					
≤35	204 (23.7)	107 (24.9)	97 (22.6)	1.051	0.591
35-45	264 (30.7)	134 (31.2)	130 (30.2)		
>45	392 (45.6)	189 (44.0)	203 (47.2)		
**Working_years**					
≤5	182 (21.2)	96 (22.3)	86 (20.0)	0.800	0.670
5-15	266 (30.9)	129 (30.0)	137 (31.9)		
>15	412 (47.9)	205 (47.7)	207 (48.1)		
**Educational degree**					
Junior high school and below	494 (57.4)	242 (56.3)	252 (58.6)	0.759	0.684
High school, secondary vocational school, technical secondary school, college	334 (38.8)	173 (40.2)	161 (37.4)		
Bachelor degree or above	32 (3.7%)	15 (3.5)173	17 (4.0%)		
**Type of work**					
First-line work	749 (87.1)	376 (87.4)	373 (86.7)	0.093	0.760
management	111 (12.9)	54 (12.6)	57 (13.3)		
**Working_shift**					
Day shift	241 (28.0)	125 (29.1)	116 (27.0)	3.606	0.307
Two shifts	187 (21.7)	89 (20.7)	98 (22.8)		
Three shifts	423 (49.2)	209 (48.6)	214 (49.8)		
Four shifts	9 (1.0)	7 (1.6)	2 (0.5)		
**Annual_income**					
≤60000	619 (72.0)	315 (73.3)	304 (70.7)	0.698	0.404
>60000	241 (28.0)	115 (26.7)	126 (29.3)		
**BMI**					
Low	8 (0.9)	2 (0.5)	6 (1.4)	9.027	0.029
Normal	388 (45.1)	214 (49.8)	174 (40.5)		
Overweight	383 (44.5)	178 (41.4)	205 (47.7)		
Obesity	81 (9.4)	36 (8.4)	45 (10.5)		
**Smoke**					
Yes	377 (43.8)	205 (47.7)	172 (40.0)	5.143	0.023
No	483 (56.2)	225 (52.3)	258 (60.0)		
**Drink**					
Yes	346 (40.2)	179 (41.6)	167 (38.8)	0.651	0.420
No	514 (59.8)	251 (58.4)	262 (61.2)		

### 3.2. Prevalence of MSDs

The results showed that the prevalence of MSDs among the underground coal miners was 55.3%, 51.2%, and 41.9% since joining the workforce, in the past year, and in the past week, respectively. The highest prevalence of MSDs was in the lower back (45.8% vs. 38.8% vs. 33.7%) and the lowest prevalence was in the hip and buttock (13.3% vs. 11.4% vs. 9.1%). All differences were statistically significant (*p* < 0.05) Table 2.

**Table 2:** Comparison of prevalence of MSDs among underground coal miners [*n* (%)].

Body parts	Since joining the work(%)	Last year(%)	Last week(%)
Neck	138 (32.1)	120 (27.9)	102 (23.7)
Shoulder	128 (29.8)	109 (25.3)	88 (20.5)
Back	94 (21.9)	87 (20.2)	75 (17.4)
Elbow	76 (17.7)	65 (15.1)	59 (13.7)
Waist	197 (45.8)	167 (38.8)	145 (33.7)
Wrist	76 (17.7)	70 (16.3)	60 (14.0)
Hips and buttocks	57 (13.3)	49 (11.4)	39 (9.1)
Knee	133 (30.9)	122 (28.4)	90 (20.9)
Ankle foot	77 (17.9)	68 (15.8)	51 (11.9)
χ^2^	192.986	150.637	128.176
*P-*value	<0.001	<0.001	<0.001

### 3.3. Occupational tension, burnout, and depression

The mean total scores of occupational tension, burnout, and depression among underground coal mine workers were 1.55 ± 0.64, 51.52 ± 11.53, and 13.83 ± 14.27, respectively. Occupational tension scores were statistically different (*p* < 0.05) between the scores of underground coal mine workers of different ages and job types: those aged 45 years and older had higher occupational tension scores than younger workers, and managers had higher occupational tension scores than front-line workers. The differences in burnout scores were statistically significant (*p* < 0.05) among coal miners in terms of age, experience, education, job type, shift situation, and economic income. Specifically, the scores of workers 35–45 years old and those >45 years old were higher than those under 35 years old; the scores of those with >15 years of experience were higher than those with ≤5 years and those with 5–15 years of experience; the scores of workers with a bachelor’s degree or above were lower than the other two groups. Moreover, the scores of first-line workers were higher than those of managers, and the scores of the two-shift and three-shift groups were higher than those of the fixed-day shift. Depression scores were statistically significant (*p* < 0.05) among coal miners of different ages and job types, and the differences were all statistically significant (*p* < 0.05): i.e., those >45 years old scored higher than those under 35 years old; those with >15 years of experience scored higher than the those with ≤5 years of experience and those with 5–15 years of experience; and first-line workers scored higher than managers Table 3.

**Table 3:** Occupation stress, occupation job burnout and depression scores among underground coal miners (X ± S).

Factors	Train	Occupational stress	Occupational job burnout	Depressed
**Age(years)**				
≤35	107	1.72 ± 0.75	47.83 ± 10.14	11.25 ± 12.36
35–45	134	1.62 ± 0.63	51.46 ± 11.22^1^	12.99 ± 14.62
>45	189	1.40 ± 0.55^2,3^	53.66 ± 12.00^2^	15.88 ± 14.78^2^
*t*/*F*		9.987	9.045	3.995
*p-*value		<0.001	<0.001	0.019
**Working_years**				
≤5	96	1.52 ± 0.63	47.75 ± 9.93	11.32 ± 12.44
5–15	129	1.66 ± 0.71	49.65 ± 11.90	12.53 ± 13.18
>15	205	1.49 ± 0.59	54.46 ± 11.28^2,3^	15.81 ± 15.46^2,3^
*t*/*F*		2.676	14.313	4.054
*p-*value		0.070	<0.001	0.018
**Educational degree**				
Junior high school and below	242	1.53 ± 0.66	51.40 ± 11.87	13.577 ± 14.63
High school, secondary vocational school, technical secondary school, college	173	1.56 ± 0.62	52.46 ± 10.84	14.84 ± 14.00
Bachelor degree or above	15	1.79 ± 0.43	42.67 ± 10.51^2,3^	6.33 ± 8.33
*t*/*F*		1.232	5.100	2.561
*p-*value		0.293	0.006	0.078
**Type of work**				
First-line work	376	1.52 ± 0.62	52.18 ± 11.40	14.81 ± 14.67
Management	54	1.77 ± 0.73	46.93 ± 11.53	6.98 ± 8.32
*t*/*F*		−2.696	3.164	5.748
*p-*value		0.007	0.002	<0.001
**Working_shift**				
Day shift	125	1.57 ± 0.68	50.78 ± 11.61	14.78 ± 15.12
Two shifts	89	1.58 ± 0.74	54.29 ± 11.93^1^	15.99 ± 14.04
Three shifts	209	1.53 ± 0.58	50.99 ± 11.08^2^	12.49 ± 13.55
Four shifts	7	1.35 ± 0.53	45.43 ± 14.26	9.29 ± 19.10
*t*/*F*		0.419	2.717	1.727
*p-*value		0.740	0.044	0.161
**Annual_income**				
≤60,000	315	1.55 ± 0.63	51.80 ± 12.04	13.78 ± 14.27
>60,000	115	1.54 ± 0.67	50.77 ± 10.03	13.97 ± 14.31
*t*/*F*		0.192	0.893	−0.120
*p-*value		0.848	0.373	0.904
**BMI**				
Low	2	1.55 ± 0.29	40.00 ± 22.63	3.00 ± 4.24
Normal	214	1.52 ± 0.62	51.33 ± 11.37	13.89 ± 14.74
Overweight	178	1.57 ± 0.68	52.13 ± 11.88	14.03 ± 13.84
Obesity	36	1.59 ± 0.60	50.28 ± 10.19	13.06 ± 14.02
*t*/*F*		0.259	0.993	0.431
*p-*value		0.855	0.396	0.731
**Smoke**				
Yes	205	1.54 ± 0.66	51.76 ± 11.07	14.58 ± 14.54
No	225	1.55 ± 0.63	51.31 ± 11.96	13.14 ± 14.01
*t*/*F*		−0.224	0.403	0.348
*p-*value		0.823	0.687	0.297
**Drink**				
Yes	179	1.51 ± 0.62	52.26 ± 10.54	13.58 ± 14.07
No	251	1.57 ± 0.66	50.99 ± 12.18	14.01 ± 14.43
*t*/*F*		−1.010	1.127	−0.310
*p-*value		0.313	0.261	0.757

### 3.4. Annual prevalence of MSDs among underground coal miners

The results of the analysis of the training set data showed that the differences in the annual prevalence of MSDs between different coal miners in terms of age, shift work, annual income, and burnout were statistically significant (*p* < 0.05). The prevalence of MSDs was higher among those >45 years old (49.5%), length of service of >15 years (56.4%), annual income <$60,000 (79.1%), and moderate burnout (43.2%) Table 4.

**Table 4:** Annual prevalence of MSDs among underground coal miner [*n* (%)].

Factors	Train	WSDs(%)		*χ^2^*	*p-*value
		Yes(*n* = 220)	No(*n* = 210)		
**Age(years)**					
≤35	107	42 (19.1)	65 (31.0)	9.286	0.010
35–45	134	69 (31.4)	65 (31.0)		
>45	189	109 (49.5)	80 (38.1)		
**Working_years**					
≤5	96	27 (12.3)	69 (32.9)	27.805	<0.001
5–15	129	69 (31.4)	60 (28.6)		
>15	205	124 (56.4)	81 (38.6)		
**Educational degree**					
Junior high school and below	242	114 (51.8)	128 (61.0)	3.729	0.155
High school, secondary vocational school, technical secondary school, college	173	97 (44.1)	76 (36.2)		
Bachelor degree or above	15	9 (4.1)	6 (2.9)		
**Type of work**					
First-line work	376	191 (86.8)	185 (88.1)	0.160	0.690
Management	54	29 (13.2)	25 (11.9)		
**Working_shift**					
Day shift	125	61 (27.7)	64 (30.5)	6.177	0.103
Two shifts	89	52 (23.6)	37 (17.6)		
Three shifts	209	101 (45.9)	108 (51.4)		
Four shifts	7	6 (2.7)	1 (0.5)		
**Annual_income**					
≤60,000	315	174 (79.1)	141 (67.1)	7.829	0.005
>60,000	115	46 (20.9)	69 (32.9)		
**BMI**					
Low	2	1 (0.5)	1 (0.5)	2.800	0. 423
Normal	214	101 (45.9)	113 (53.8)		
Overweight	178	99 (45.0)	79 (37.6)		
Obesity	36	19 (8.6)	17 (8.1)		
**Smoke**					
Yes	205	100 (45.5)	105 (50.0)	0.890	0.346
No	225	120 (54.5)	105 (50.0)		
**Drink**					
Yes	179	98 (44.5)	81 (38.6)	1.578	0.209
No	251	122 (55.5)	129 (61.4)		
**Occupational stress**					
<1	51	25 (11.4)	26 (12.4)	3.152	0. 207
=1	14	4 (1.8)	10 (4.8)		
>1	365	191 (86.8)	174 (82.9)		
**Occupational job burnout**					
No	27	15 (6.8)	12 (5.7)	20.747	<0.001
Mild	195	82 (37.3)	113 (53.8)		
Moderate	174	95 (43.2)	79 (37.6)		
Severe	34	28 (12.7)	6 (2.9)		
**Depression**					
No	273	140 (63.6)	133 (63.3)	0.416	0.937
Mild	21	12 (5.5)	9 (4.3)		
Moderate	33	17 (7.7)	16 (7.6)		
Severe	103	51 (23.2)	52 (24.8)		

### 3.5. Binary logistic regression of the prevalence of MSDs

**Figure 1:**
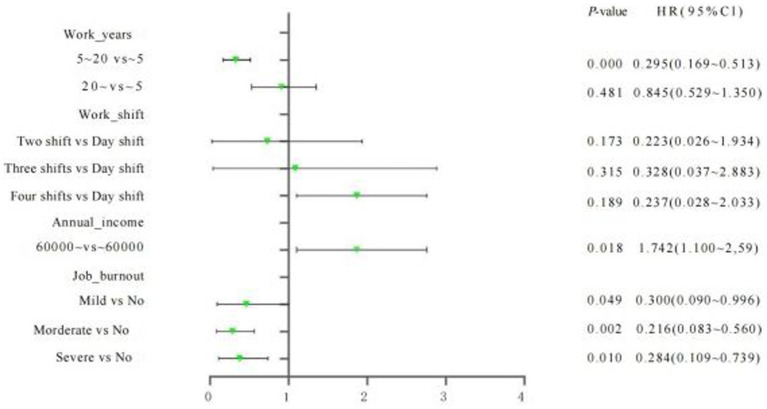
Forest graph of selected factors.

Binary logistic regression analysis with the occurrence of MSDs as the dependent variable and the factors with significant differences (*p* < 0.05) in the univariate analysis in Table 4 as the independent variables revealed that the prevalence of MSDs was lower among underground coal mine operation workers with 5–20 years of service compared to those with <5 years of service (OR = 0.295, 95% CI: 0.169–0.513); the prevalence of MSDs was higher in coal miners with an annual income of >$60,000 compared to those with an annual income of ≤$60,000 (OR = 1.742, 95% CI: 1.100–2.759); MSD prevalence was lower in those with severe burnout compared to those with zero burnout (OR = 0.284, 95% CI: 0.109–0.739; Table 5; Figure 1).

**Table 5:** Risk predictors for MSDs for underground coal miners.

	Comparison group	control group	β	SE	Wald χ^2^	*P-*value	OR(95%*CI*)
Intercept			0.039	0.179	0.047	0.829	1.039
Age(years)	35–45	≤35	−0.154	0.304	0.257	0.612	0.857 (0.473~1.555)
	>45		−0.160	0.242	0.438	0.508	0.852 (0.530~1.369)
Working_years	5–20	≤5	−1.167	0.323	13.092	0.000	0.311 (0.165~0.586)
	>20		−0.21	0.251	0.232	0.630	0.886 (0.542~1.450)
Annual_income	>60,000	≤60,000	0.615	0.233	6.940	0.008	1.849 (1.170~2.922)
Occupational job burnout	Mild	No	−1.164	0.608	3.661	0.056	0.312 (0.095~1.029)
	Moderate		−1.523	0.485	9.848	0.002	0.218 (0.084~0.565)
	Severe		−1.206	0.485	6.177	0.013	0.299 (0.116~0.775)

### 3.6. Construction and validation of nomogram model

**Figure 2:**
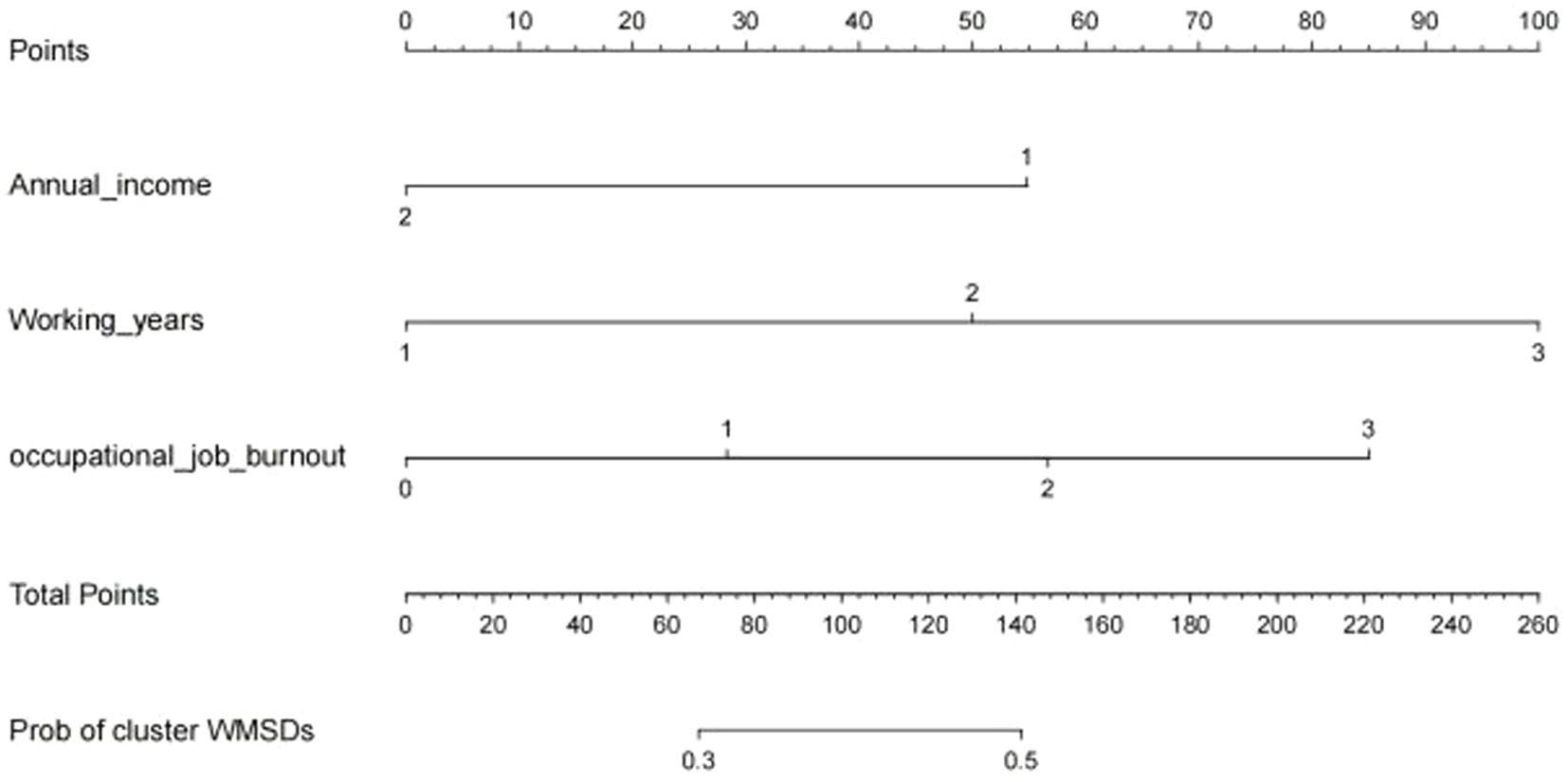
Nomogram for predicting the risk of MSDs in underground coal miners.

**Figure 3:**
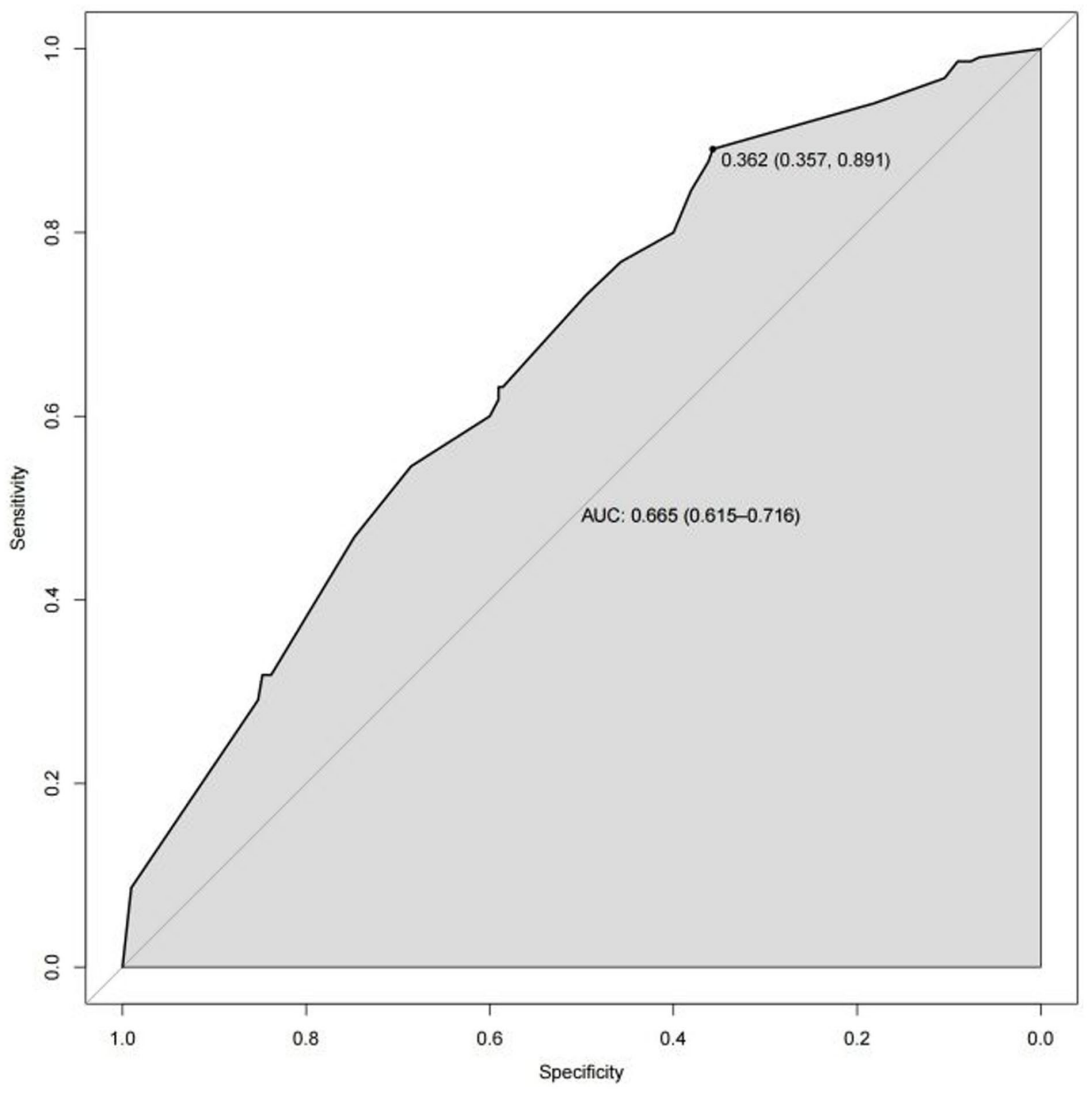
ROC curve of nomogram model for predicting the risk of MSDs in underground coal miners from the training set.

**Figure 4:**
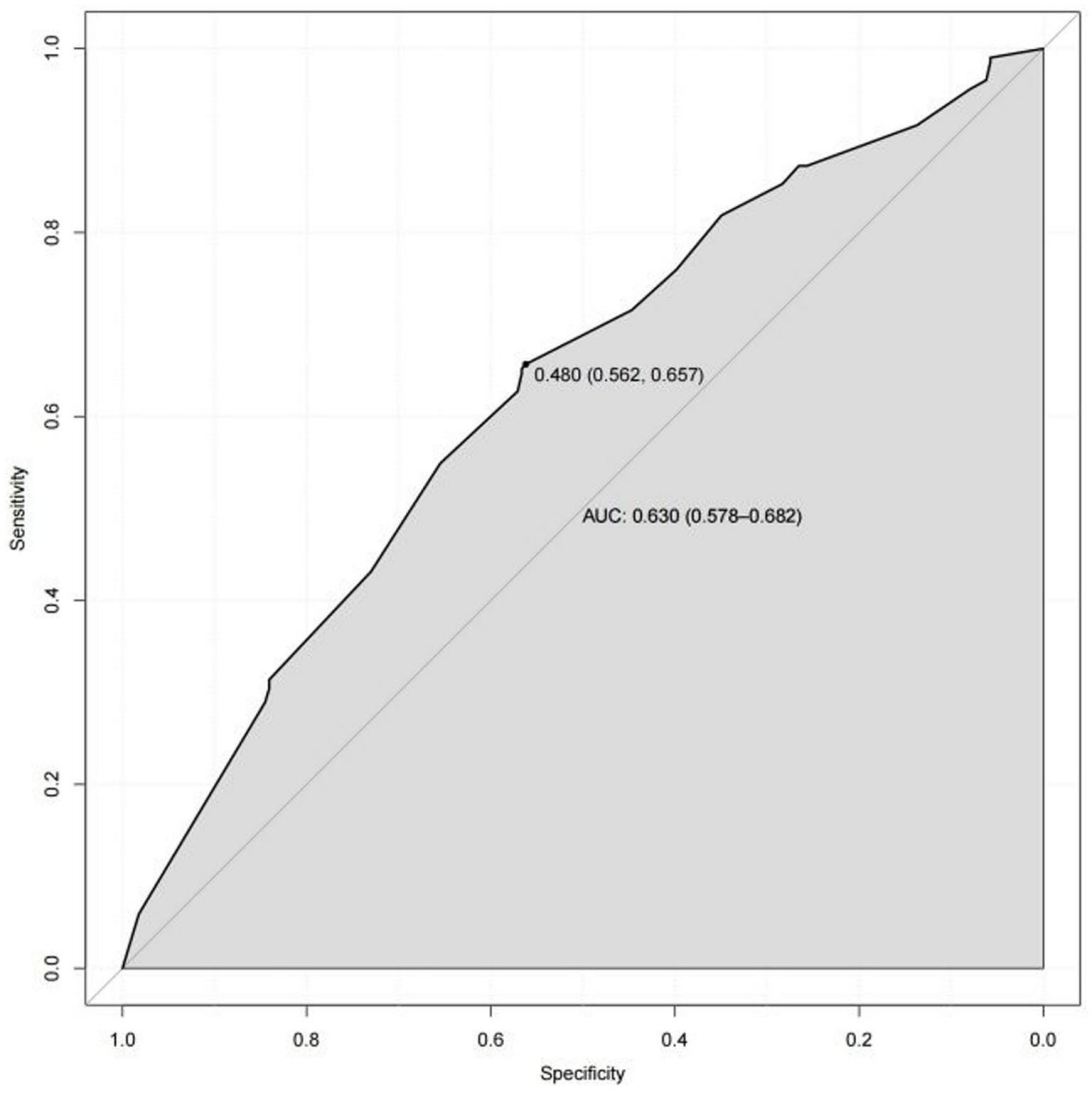
ROC curve of nomogram model for predicting the risk of MSDs in underground coal miners from the validation set.

**Figure 5:**
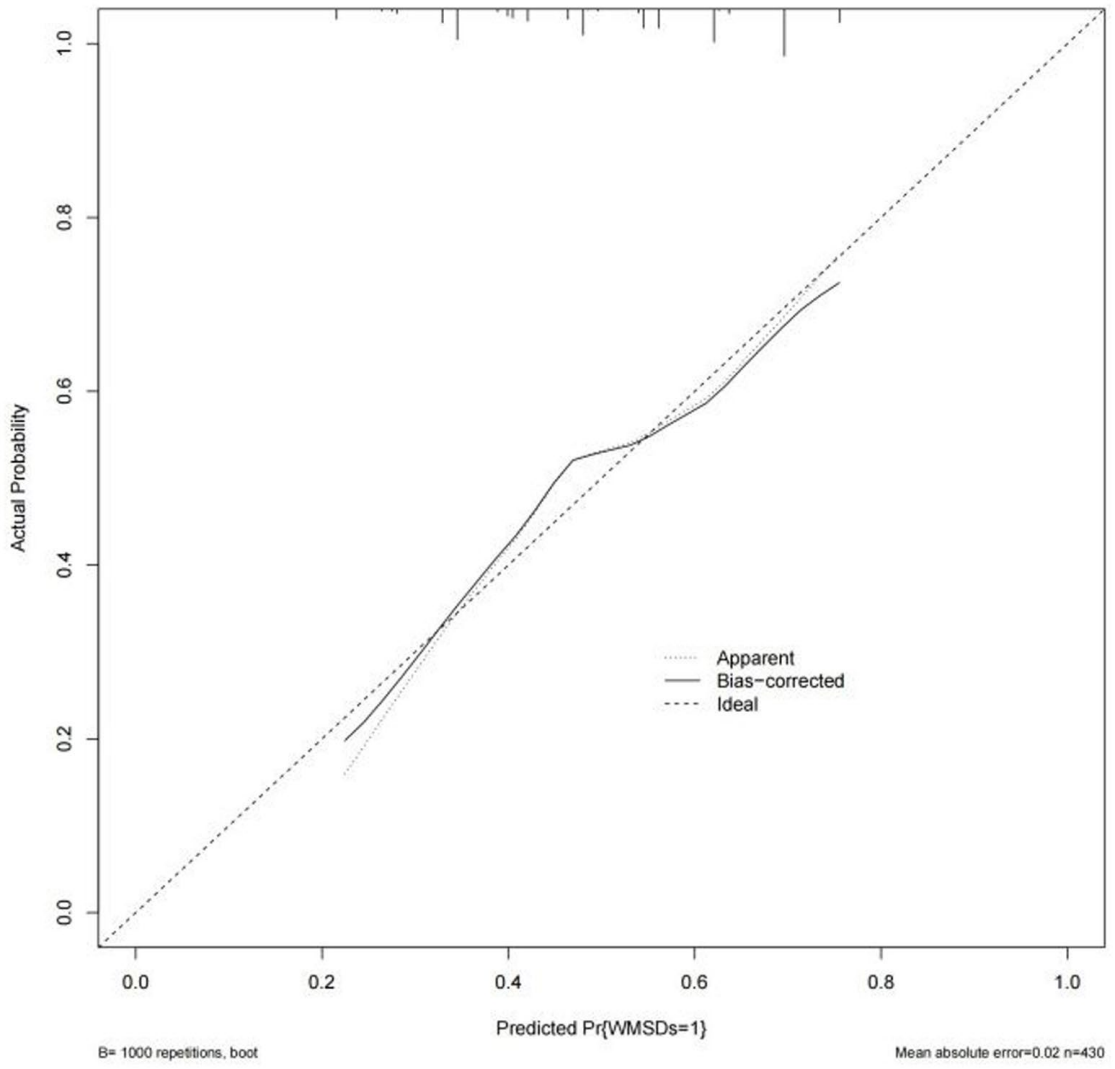
Calibration curve of nomogram model for predicting the risk of MSDs in underground coal miners from the training set.

**Figure 6:**
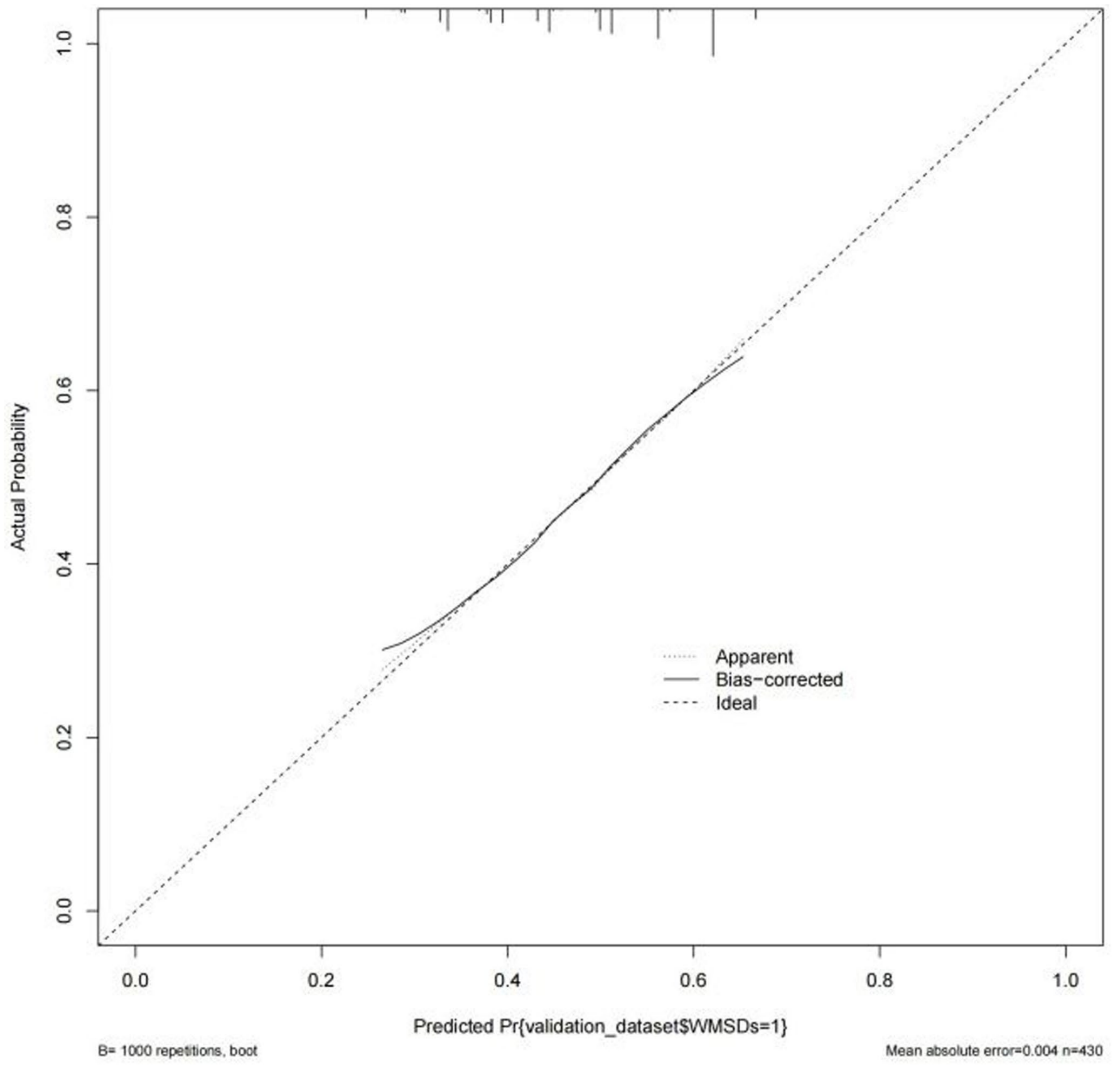
Calibration curve of nomogram model for predicting the risk of MSDs in underground coal miners from the validation set.

**Figure 7:**
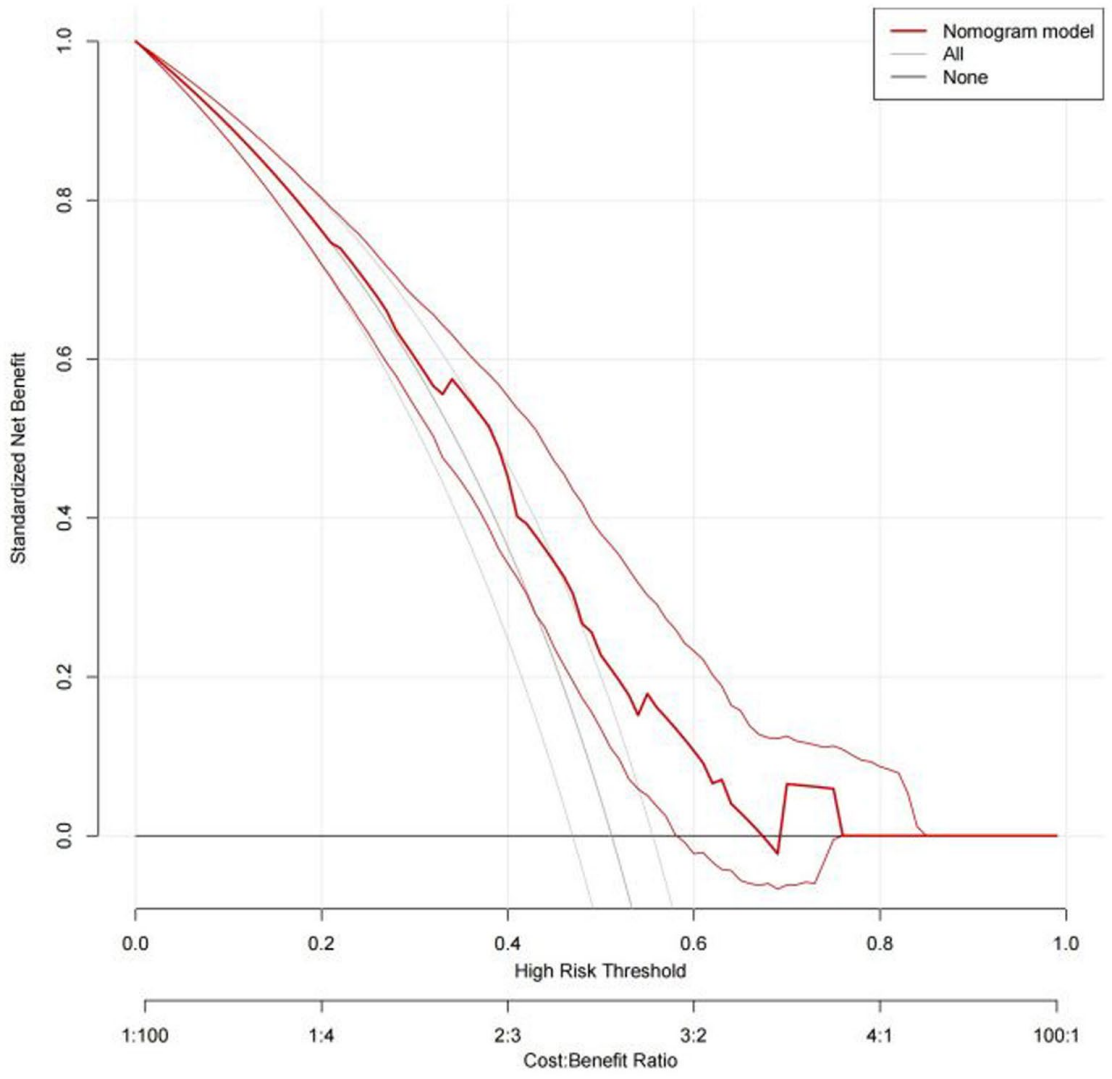
DCA curve of nomogram model for predicting the risk of MSDs in underground coal miners from the training set.

**Figure 8:**
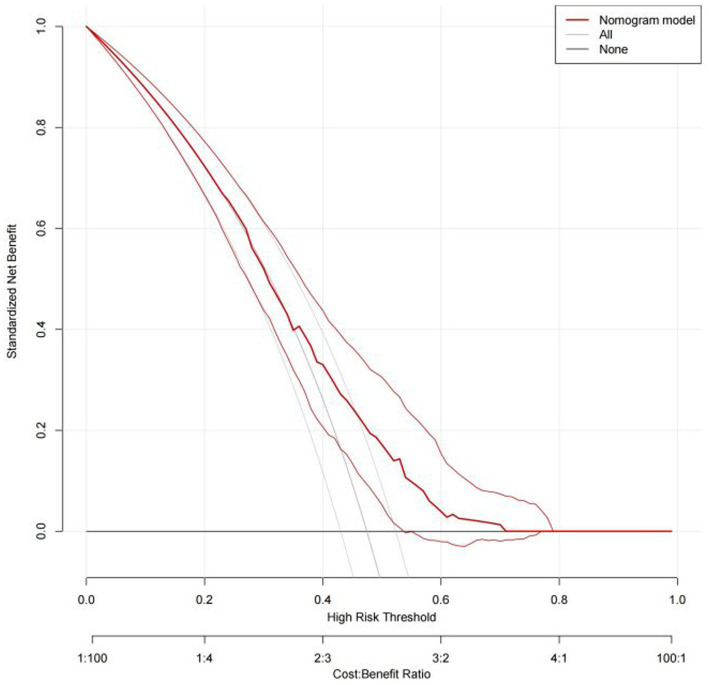
DCA curve of nomogram model for predicting the risk of MSDs in underground coal miners from the validation set.

Based on the results of multi-factor logistic regression analysis, the obtained independent predictors were incorporated into the prediction model in order to build an individualized nomogram graph prediction model for MSDs in underground coal mine workers (Figure 1). For example, for coal miners with 2 years of service, an annual income of >$60,000, and moderate burnout, the probability of having MSDs is approximately 0.42. After external validation, the nomogram model was found to have good discriminatory power, with the area under the work characteristic curve (AUC) of 0.665 (95% CI: 0.615–0.716) and 0.630 (95% CI: 0.578–0.682) for the training set and validation set, respectively. According to the calibration curve results, the prediction probability of the training set was close to the standard curve, with an accuracy of 0.90 and a mean absolute error of 0.02. The prediction probability of the validation set was close to the standard curve, with an accuracy of 0.80 and a mean absolute error of 0.004, suggesting good calibration and discrimination of the model. The DCA curve suggested that model had good predictive value for the predictive value of MSDs (Figures 2–8).

## 4. Discussion

With the accelerated pace of life, people are suffering from stress from many aspects of family, work, and life. These factors seriously threaten the mental health of occupational populations. Occupational psychological problems (occupational stress, burnout, depression, etc.) in occupational populations have become the focus of research in recent years (9–11). The top three occupational groups with the highest standardized prevalence of MSDs are aircrew, medical personnel, and vegetable shed workers, in that order (12). Previous studies found that the prevalence of MSDs in Chinese airline crew members was 86% (13), 47.4%–85.5% for medical personnel (14, 15), 84.04% for vegetable shed workers (16), and 65.58% for coal miners (9). In the present study, the prevalence of MSDs among underground coal mine workers was found to be 55.3%, 51.2%, and 41.9% since joining the workforce, in the last year, and in the last week, respectively. Our results are slightly lower than the results of previous studies (17), and this difference may be because our study included only male samples. Low back pain was the main site of pain in MSDs among coal miners in different periods, which is consistent with the findings of Dong et al. (18). Because of the complex occupational hazards involved in coal mining, the posture of standing, lifting, squatting, or sitting during the operation can cause tension, discomfort, or pain in the lumbar region. Thus, coal miners mainly present with lumbar pain.

Coal miners are prone to occupational psychological problems such as burnout, which can lead to alienation from relatives and friends, and to extreme behavior (19). Psychological factors often increase the prevalence of chronic diseases and psychological disorders, and they have a great impact on the physical and mental health and work efficiency of underground coal miners (20–22). The occupational stress states of medical personnel and firefighters lead to higher symptoms of MSDs (23, 24). The results of the current study did not indicate any effect of occupational stress on MSDs, which is consistent with the findings of Indian scholars (25). Therefore, the effect of the ERI on MSDs in coal miners needs further validation (26). Studies have found that burnout can increase the prevalence of hypertension, anxiety, and depression, which can also lead to the development of MSDs (27, 28). Therefore, psychological guidance for coal miners may reduce the occurrence of MSDs.

We found that length of service, annual income, and burnout were all associated with the prevalence of MSDs among coal miners. Compared with those with <5 years of service, coal miners with 5–20 years of service had a lower prevalence of MSDs. This may be due to the fact that with more experience, workers find a suitable working position, effectively reducing the prevalence of MSDs. We also found that workers with a higher annual income worked more, and had a higher prevalence of MSDs. Compared with coal miners with zero burnout, the prevalence of MSDs was higher in those with mild, moderate, and severe burnout. Burnout had a lower prevalence of MSDs, which is inconsistent with previous results (29, 30) but consistent with the results of Ge Hua’s study (31). This may be due to the fact that burned-out coal miners are less motivated to work, resulting in fewer activations of the musculoskeletal system and thus protecting them from developing MSDs.

In this study, a nomogram was established based on the results of binary logistic regression analysis to obtain three independent predictors (i.e., years of service, annual income, and burnout). These factors can be used to determine the probability of the corresponding risk of developing MSDs among coal miners by calculating the total score of the risk factors. Compared with other predictive models, the nomogram can provide better, personalized prediction and risk assessment in a more intuitive and visual way. This study has good efficacy in terms of discrimination, calibration, and clinical benefit, indicating that the nomogram constructed in this study is important for analyzing the risk of developing MSDs in underground coal miners.

## 5. Conclusion

This study found that a nomogram established by three predictors of work experience, annual income, and burnout status has potential clinical application regarding the prevalence of MSDs among workers in underground coal mines. Future multicenter trials are still needed to verify the reliability and applicability of the model.

### 5.1. Theoretical and practical implications of the study

The problem of MSDs among underground workers in coal mines is of concern, due to the specificity of the operating environment. Our study monitored the occupational psychological data of underground workers in coal mines and explored the influence of occupational psychology on the prevalence of MSDs. We analyzed the occupational psychological data of underground coal mine workers in Xinjiang, identified the independent predictors of MSDs among underground coal mine workers, and constructed a prediction model of MSDs through the nomogram to provide a theoretical basis for the implementation of related policies at a later stage.

### 5.2. Strengths and limitations of this study

The model was developed based on data regarding the occupational psychology and prevalence of MSDs in 860 study participants, to effectively identify potential occupational health risk factors.

(1) Innovation: To our knowledge, this study was the first to use a nomogram to predict the prevalence of MSDs among underground workers in coal mines. (2) The risk of MSDs can be predicted individually for each underground coal mine worker. (3) This study was a cross-sectional study, and it was not possible to determine the causal relationship between occupational psychology and the occurrence of MSDs. (4) The study was limited to coal miners in Xinjiang. If possible, a multicenter trial should be conducted to refine this model.

## Data availability statement

The original contributions presented in this study are included in the article/Supplementary material, further inquiries can be directed to the corresponding author.

## Ethics statement

Written informed consent was obtained from the individual(s) for the publication of any potentially identifiable images or data included in this article.

## Author contributions

LN and HZ developed the research plan. HD, WL, YY, XL, and LS collected and managed the data. HZ and SY completed the data analysis. HZ and LN drafted the manuscript. JL and XY checked and revised the manuscript. All authors contributed to the article and approved the submitted version.

## Funding

Funding was from the Natural Science Foundation of Xinjiang Uygur Autonomous Region (2020D01C177) and the Natural Science Foundation of Xinjiang Uygur Autonomous Region (2020D01C152) and the “14th Five-year” Key Discipline (Plateau discipline)-Public Health and Preventive Medicine (Xinjiang Uygur Autonomous Region, China).

## Conflict of interest

The authors declare that the research was conducted in the absence of any commercial or financial relationships that could be construed as a potential conflict of interest.

## Publisher’s note

All claims expressed in this article are solely those of the authors and do not necessarily represent those of their affiliated organizations, or those of the publisher, the editors and the reviewers. Any product that may be evaluated in this article, or claim that may be made by its manufacturer, is not guaranteed or endorsed by the publisher.
