# Supplementary figures and images for: Construction and validation of a musculoskeletal disease risk prediction model for underground coal miners
Source: Front Public Health. 2023 Jul 11;11:1099175. doi: 10.3389/fpubh.2023.1099175 (PMC10368395; doi:10.3389/fpubh.2023.1099175)

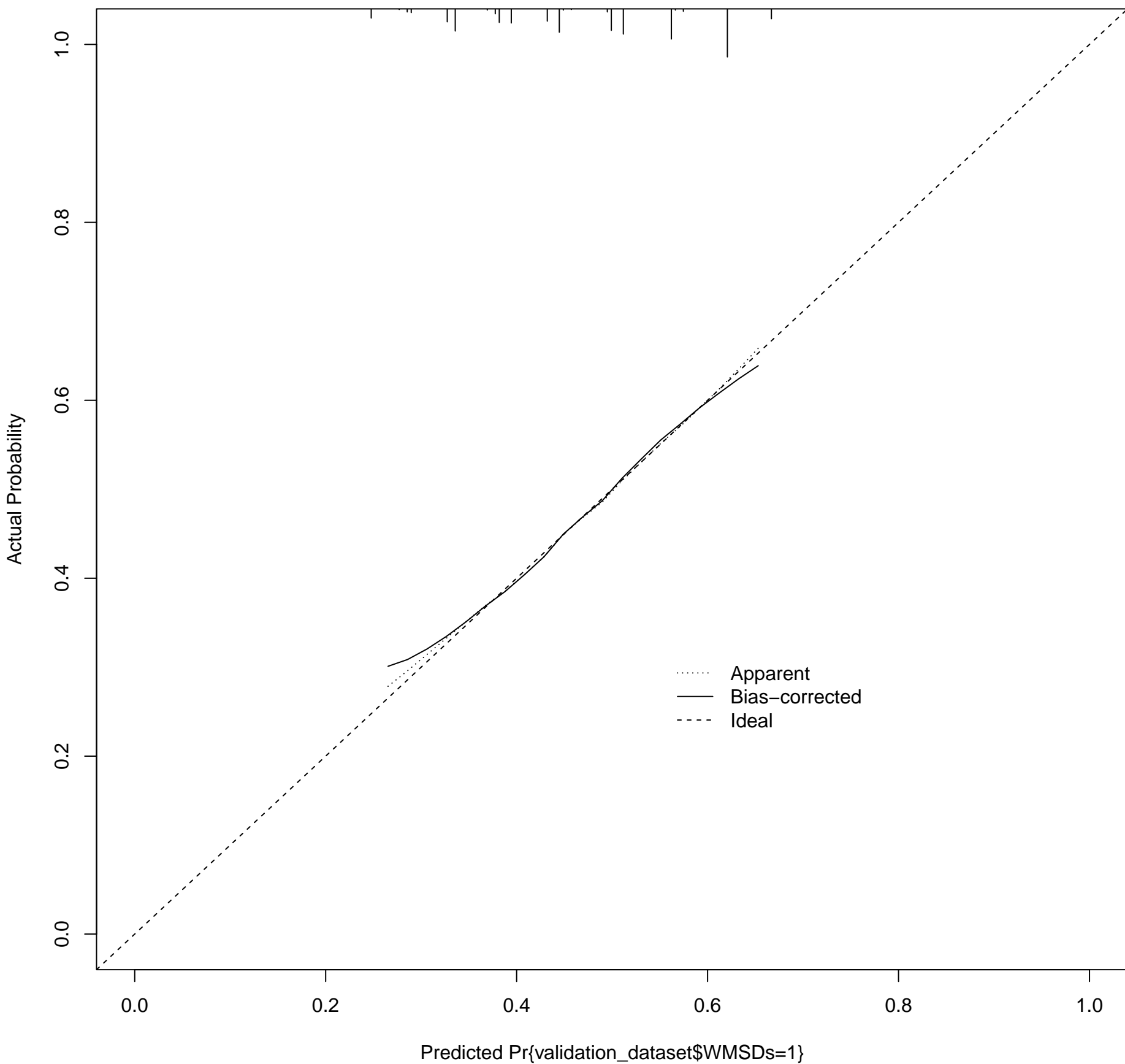

Supplement: Supplementary file 1 [file Image_1.PDF]

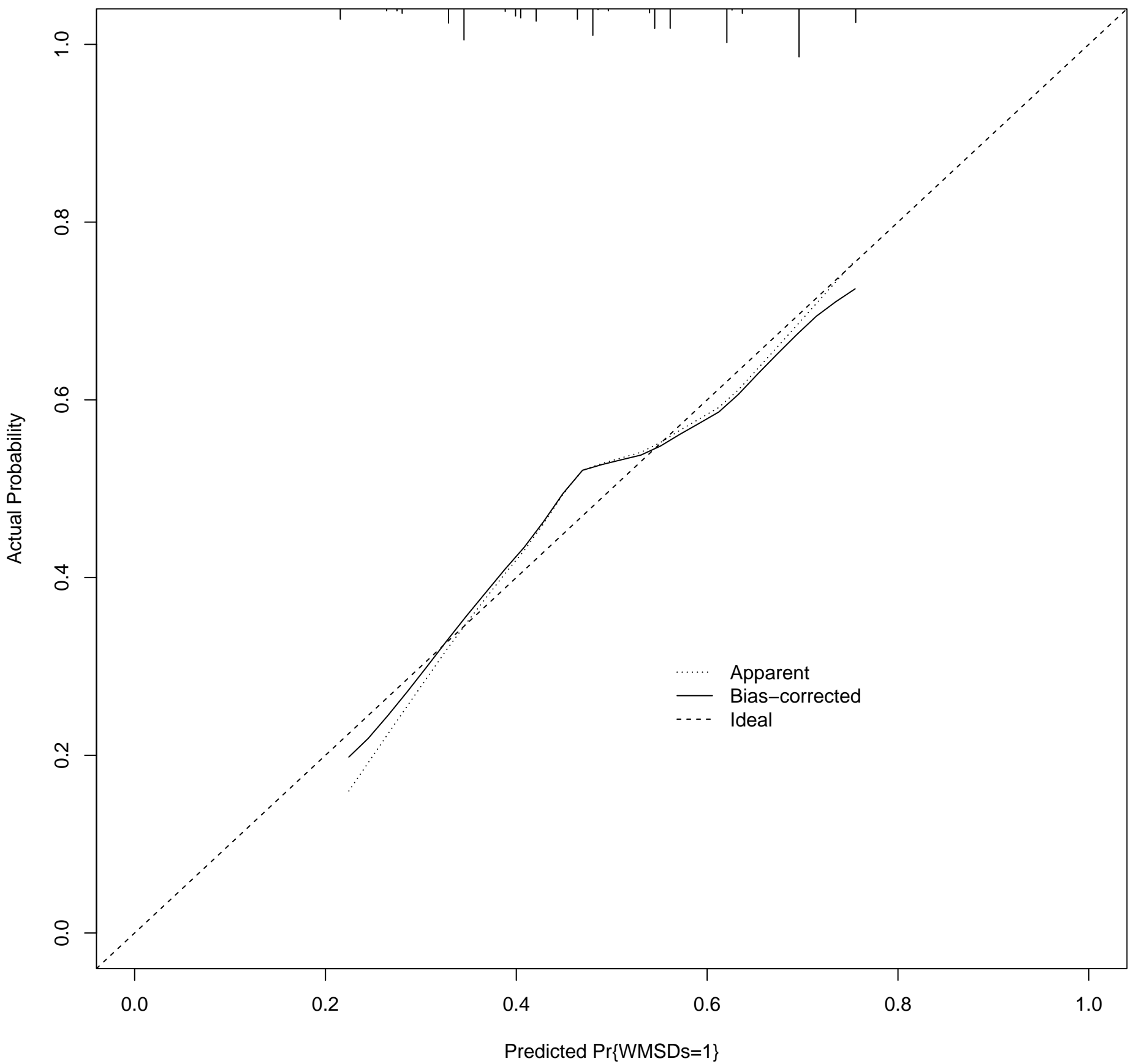

Supplement: Supplementary file 2 [file Image_2.PDF]

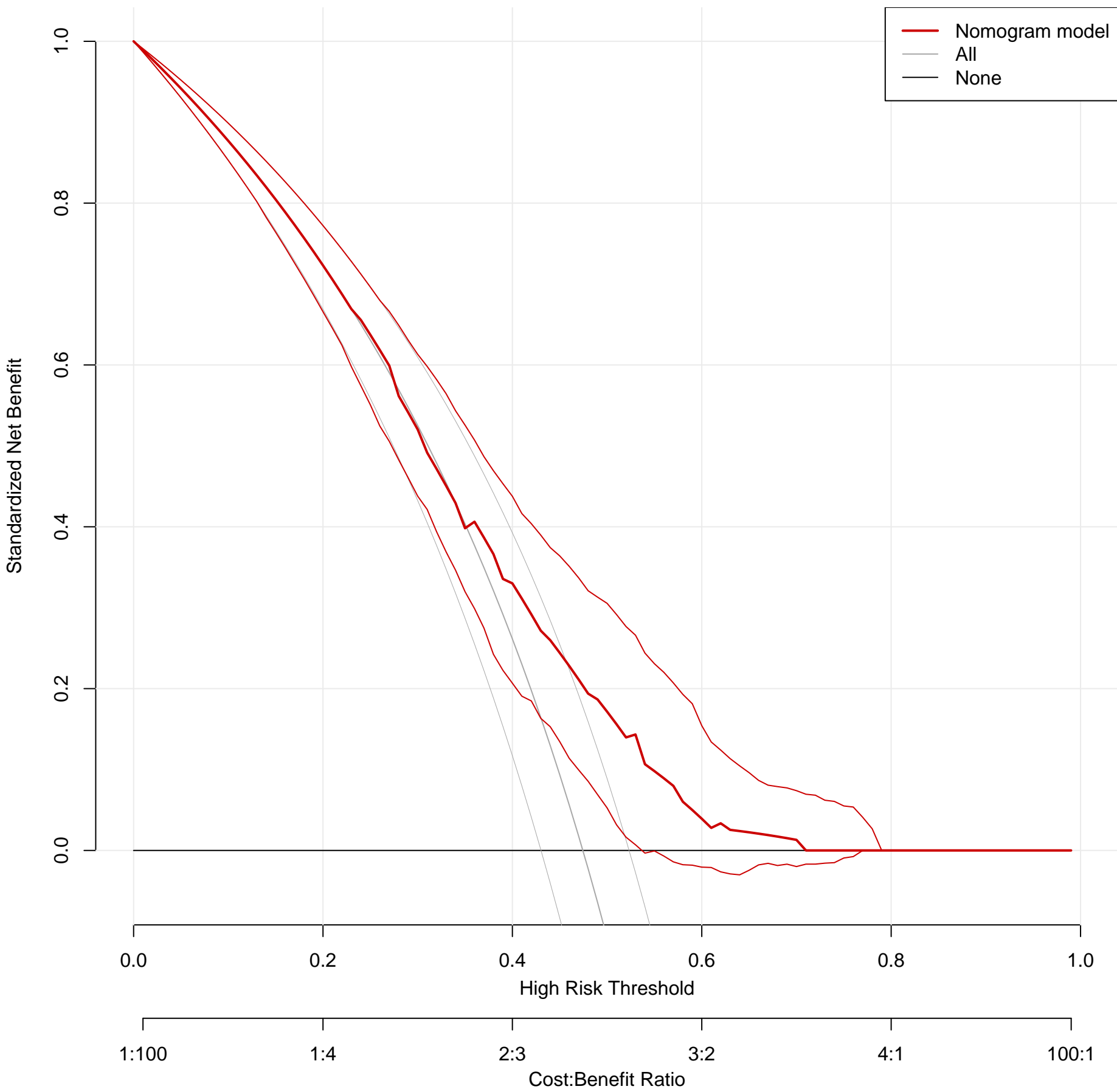

Supplement: Supplementary file 3 [file Image_3.PDF]

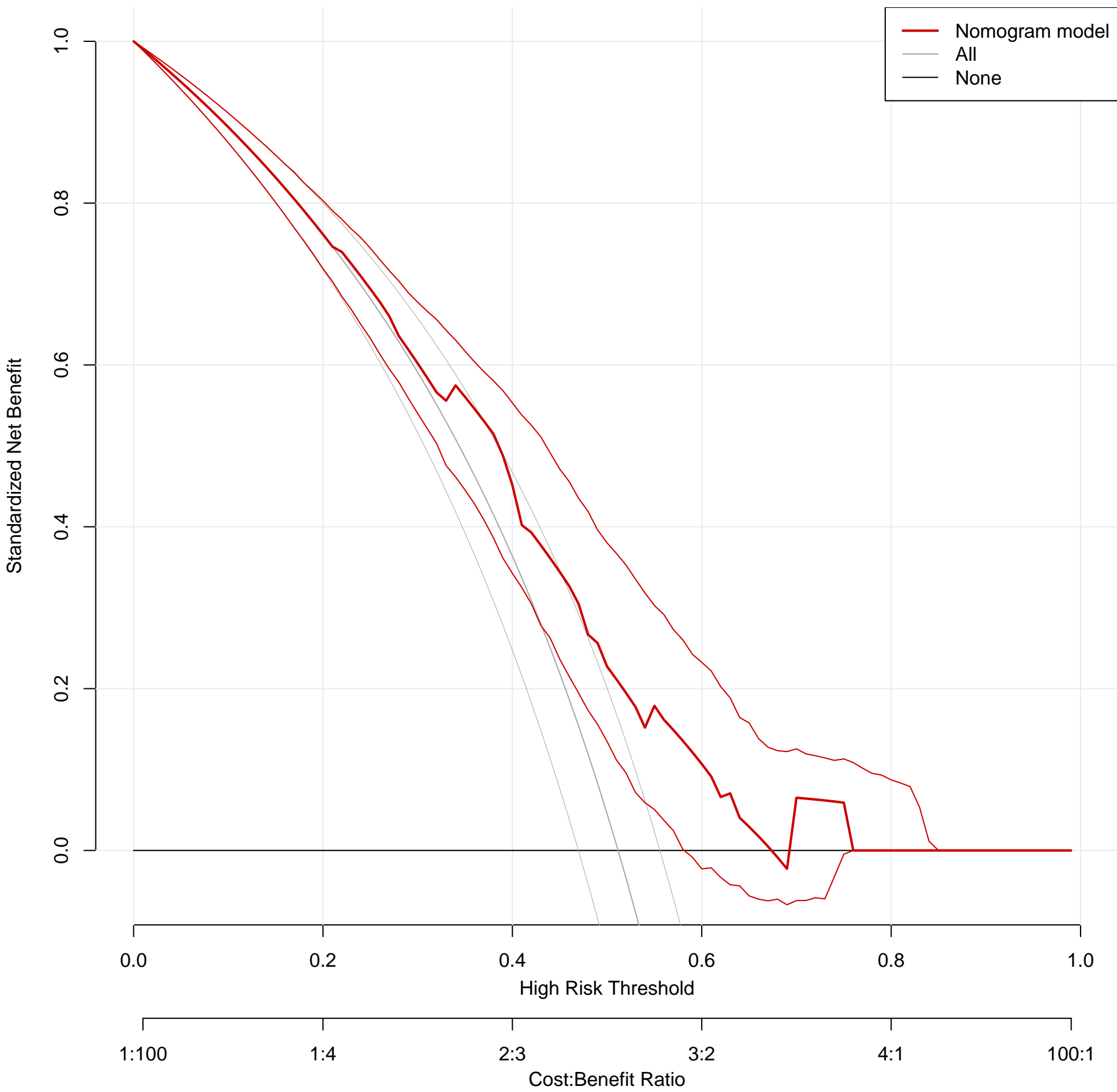

Supplement: Supplementary file 4 [file Image_4.PDF]

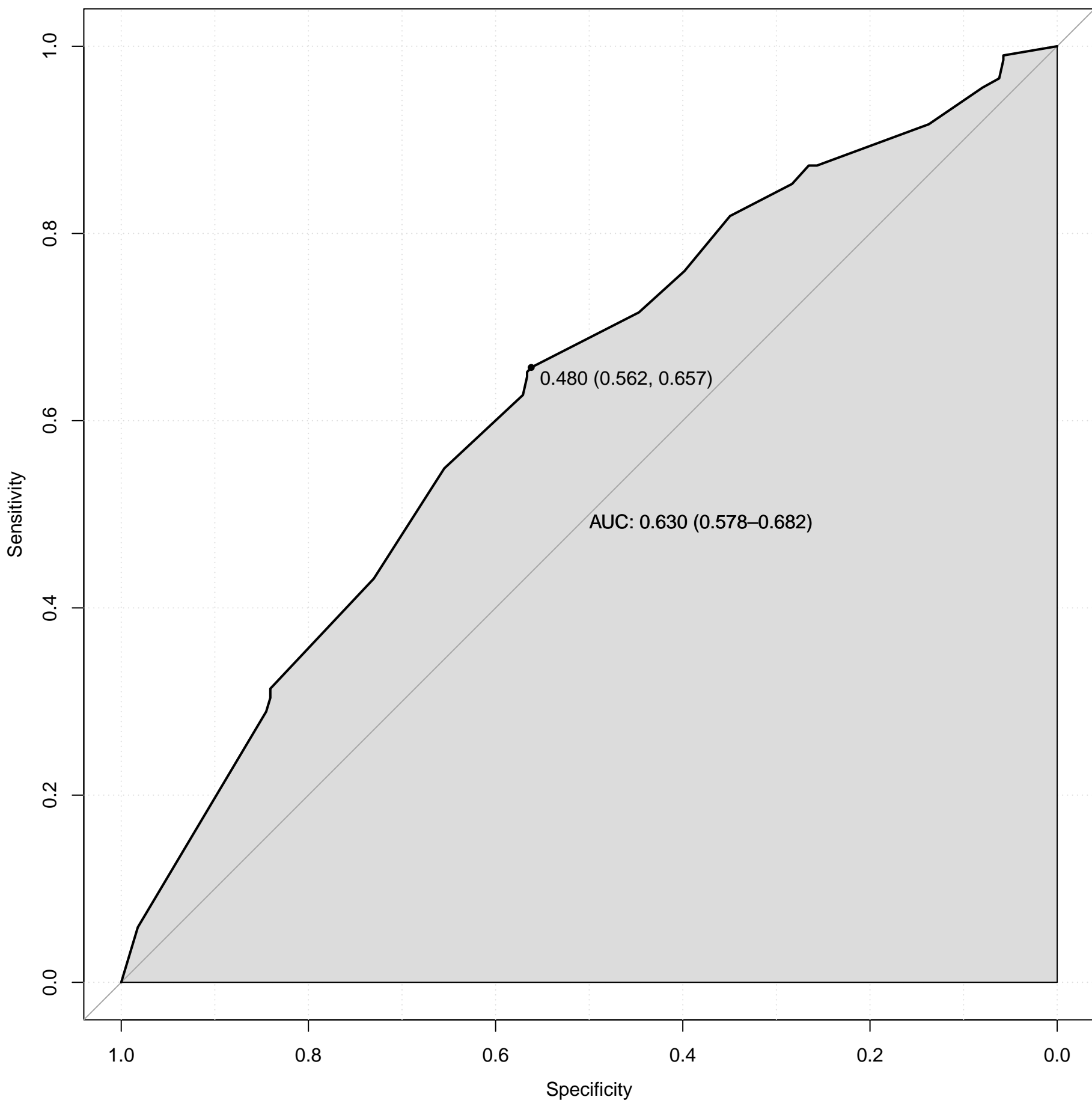

Supplement: Supplementary file 6 [file Image_6.PDF]

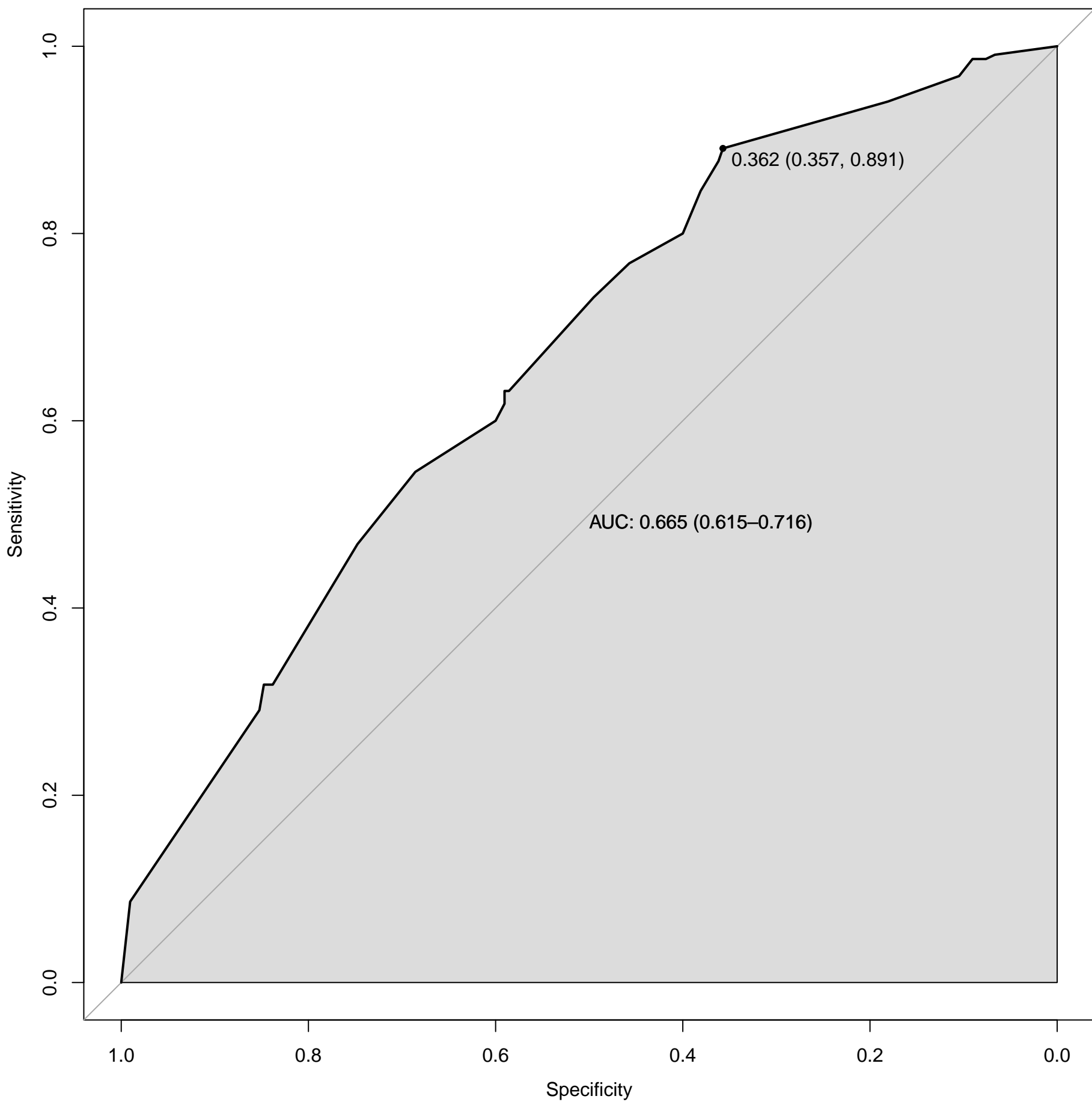

Supplement: Supplementary file 7 [file Image_7.PDF]
