# Supplementary material for: Construction and validation of a musculoskeletal disease risk prediction model for underground coal miners
Source: Front Public Health. 2023 Jul 11;11:1099175. doi: 10.3389/fpubh.2023.1099175 (PMC10368395; doi:10.3389/fpubh.2023.1099175)
Supplement: Supplementary file 5 [file Image_5.PDF]

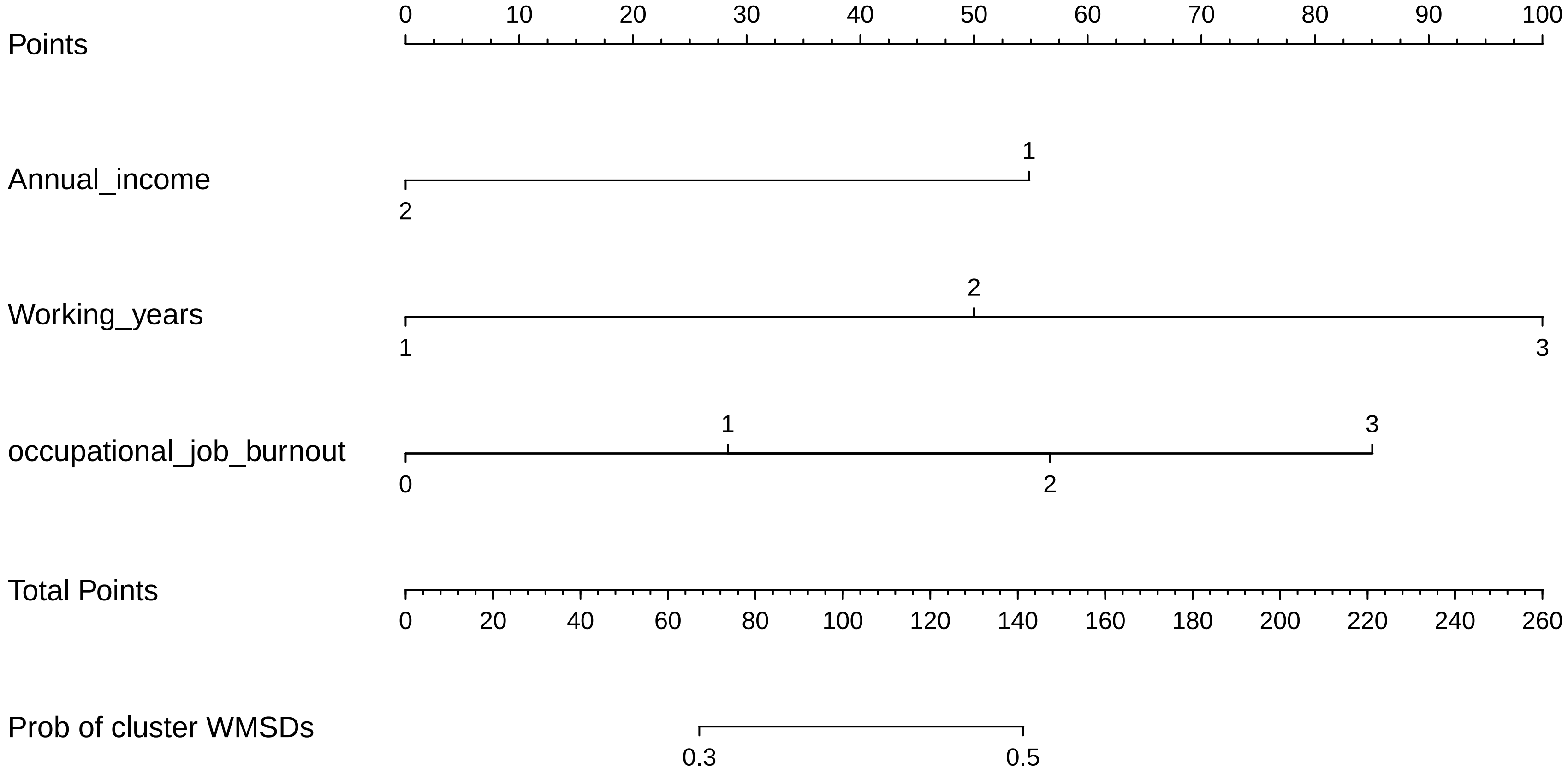
Points
0
10
20
30
40
50
60
70
80
90
100
Annual_income
1
2
Working_years
2
1
3
occupational_job_burnout
1
3
0
2
Total Points
0
20
40
60
80
100
120
140
160
180
200
220
240
260
Prob of cluster WMSDs
0.3
0.5
